# The Modified painDETECT Questionnaire for Patients with Hip or Knee Osteoarthritis: Translation into Dutch, Cross-Cultural Adaptation and Reliability Assessment

**DOI:** 10.1371/journal.pone.0146117

**Published:** 2015-12-31

**Authors:** Wietske Rienstra, Tim Blikman, Frans B. Mensink, Jos J. A. M. van Raay, Baukje Dijkstra, Sjoerd K. Bulstra, Martin Stevens, Inge van den Akker-Scheek

**Affiliations:** 1 Department of Orthopedics, University Medical Center Groningen, Groningen, The Netherlands; 2 Department of Orthopedics, Martini Hospital Groningen, Groningen, The Netherlands; 3 Department of Orthopedics, Medical Center Leeuwarden, Leeuwarden, The Netherlands; The University of Tokyo Hospital, JAPAN

## Abstract

There is a growing amount of evidence that alteration in pain processing by the peripheral and central nervous system play a role in osteoarthritis pain, leading to neuropathic-like symptoms. It is essential to identify knee and hip osteoarthritis patients with a neuropathic pain profile in order to offer such patients education and additional treatment options besides conventional pain treatment. The painDETECT Questionnaire is a self-report questionnaire developed to discriminate between nociceptive and neuropathic pain. This questionnaire was modified to fit patients suffering from knee osteoarthritis. The aim of this study was to translate and cross-culturally adapt the modified painDETECT Questionnaire to the Dutch language and to provide a modified version to fit patients with hip osteoarthritis. Reliability for internal consistency, repeatability and floor and ceiling effects were subsequently assessed. A total of 278 patients were included in the reliability study and 123 patients in the repeatability analysis. The Dutch modified painDETECT Questionnaire shows good internal consistency and small relative measurement errors, represented by a good intraclass correlation coefficient. Absolute measurement error, represented by the Standard Error of Measurement, was acceptable. However, a measurement bias might be present when it comes to repeatability. To our knowledge, this study is the first to provide a Dutch modified painDETECT Questionnaire to fit hip and knee osteoarthritis patients and to assess internal consistency, reliability and agreement. International guidelines were followed in the translation process and this study has ample sample size with an adequate time interval for repeatability. Based on this study, the Dutch modified painDETECT Questionnaire seems to be fit as a discriminative tool to identify knee and hip osteoarthritis patients with a neuropathic pain profile. Whether it is also suitable as an evaluative tool to record changes over time or after an intervention remains open to further investigation.

## Introduction

Osteoarhtritis (OA) is the most common arthritic joint disease, affecting mainly older adults [1–3]. Its worldwide symptomatic and economic burden is tremendous [1,3]. Due to increasing age of the population the burden of OA will likely become even greater in the future [3–6]. Mostly joints of the lower extremity are affected, especially the hip and knee, leading to pain and disability [1].

For most patients with OA, pain is the main reason to seek medical consultation and treatment. Over time the pain often transforms from intermittent weight-bearing to persistent, chronic pain [3,7]. The etiology of pain in OA is complex and its exact pathophysiology is still not completely clarified. A broad spectrum of mechanisms is believed to be involved, both nociceptive and neuropathic [8–10]. There is a growing amount of evidence that alteration in pain processing by the peripheral and central nervous system play a role in OA pain leading to sensitization [8–14]. Sensitization of the nervous system may lead to neuropathic-like symptoms such as allodynia, hyperalgesia and hypoesthesia [15]. Up to 19% of patients suffering from hip OA and 19–37% of patients suffering from knee OA experience possible or likely neuropathic pain localized in or around their hip or knee [15–20].

It is essential to identify knee and hip OA patients with a neuropathic pain profile in order to offer such patients education and additional treatment options besides conventional pain treatment. A reliable and valid tool for assessing neuropathic pain symptoms in OA patients is therefore of great importance. Besides diagnosis by a specialized pain physician, there are several tools to assess neuropathic pain symptoms, ranging from self-report questionnaires to a more objective tool like Quantitative Sensory Testing (QST) [21–24]. For evaluation in a non-clinical, ambulatory setting only self-report questionnaires seem feasible, as the more objective methods are time-consuming, elaborate, require trained examiners, and are therefore only applicable in a clinical setting.

The painDETECT Questionnaire (PDQ) is a self-report questionnaire that has been developed to discriminate between nociceptive and neuropathic pain. Originally it was developed for patients with chronic low back pain [25]. With a sensitivity and specificity of 85% and 80% respectively, accuracy of the PDQ is comparable to other self-report questionnaires [24,26]. Hochman et al. modified this questionnaire to fit patients suffering from knee OA. This modified painDETECT questionnaire (mPDQ) has improved upon the PDQ in content validity, face validity and feasibility for the knee OA population [18].

No Dutch-language version of the mPDQ is currently available. It should be noted that the mPDQ only considers knee OA, it is not modified to fit patients suffering from hip OA. Hence the aims of this study were: 1) to translate and cross-culturally adapt the mPDQ to the Dutch language, 2) to adjust the Dutch mPDQ to fit patients with hip OA, and 3) to assess the reliability of the Dutch mPDQ.

## Methods

This study consists of two stages. First, translation and cross-cultural adaptation of the mPDQ took place. In this stage the mPDQ was also adapted to fit patients suffering from hip OA. Secondly, internal consistency, repeatability and floor-ceiling effects were evaluated. The study was approved by the Medical Ethical Committee of University Medical Center Groningen (no. METc2014/087). Informed consent was considered obtained if the patient granted our request to participate in the study by returning a set of completed questionnaires. If patients did not want to participate in the study they were requested to return the blank set of questionnaires. Patients were informed of this way of obtaining consent by an information letter. In this information letter they were also informed of the voluntary nature of the study, and that all data was processed anonymously. The Medical Ethical Committee specifically approved this consent procedure.

### Translation and cross-cultural adaptation

The international guidelines proposed by Beaton et al. were followed during translation and cross-cultural adaptation of the mPDQ into Dutch [27]. Forward translation of the English mPDQ into Dutch was done independently by two bilingual native Dutch speakers, one with a medical background and the other a layman. Discrepancies between the two forward-translated versions of the mPDQ were subsequently identified and resolved during a consensus meeting with an expert as referee. This synthesis process was documented in a written report. Next, backward translation of the Dutch consensus version of the mPDQ into English was done independently by two bilingual native English speakers, again one with a medical background and the other a layman. These two translators had never seen the original version of the mPDQ and were neither informed nor familiar with the concept of the study. In this way, the content validity was verified. All versions were reviewed during a final consensus meeting of the two native Dutch-speaking translators and the expert. Consensus was obtained and a pre-final version of the Dutch mPDQ (mPDQ-NL) was composed. Finally, the mPDQ-NL was slightly adjusted to fit patients suffering from hip OA. The two questionnaires are identical except for substituting the target joint “knee” for “hip” in every question.

After this translation and adaptation process, comprehensibility of the pre-final mPDQ-NL was tested in a pilot study. Thirty patients suffering from knee or hip OA who had an appointment at the outpatient clinic of the Department of Orthopedic Surgery of University Medical Center Groningen were asked to complete the questionnaire. These patients were asked to report any problems filling out the questionnaire and to give their comments. This resulted in a few minor adaptations to the questionnaire like underscoring some items to enhance interpretability. See S1 Text for the final mPDQ-NL hip and knee.

### Reliability

#### Participants

Patients suffering from idiopathic hip or knee OA who were receiving conservative treatment or were on the waiting list for Total Hip or Knee Arthroplasty (THA/TKA) were eligible to participate. A random sample of 604 patients who had visited the outpatient clinic between July 2013 and May 2014 was obtained from the Departments of Orthopedic Surgery of University Medical Center Groningen, Martini Hospital Groningen and Medical Center Leeuwarden (all in the Netherlands). Exclusion criteria were: age below 18 years; hip or knee surgery in the past six months; severe neurological, cognitive or psychiatric disorders; and inadequate understanding of written Dutch.

#### Procedure

Eligible patients received an information letter, the mPDQ-NL hip or knee, a questionnaire on demographic characteristics and comorbidities, and a pre-paid reply envelope by mail. Patients were requested to complete the questionnaires. It was explained in the information letter that returning a set of completed questionnaires was considered as informed consent to participate in the study. If patients suffered from OA in more than one joint they were asked to complete the questionnaire for the hip or knee joint that was most symptomatic. To assess repeatability, a second mPDQ-NL was sent to participants after an interval of two weeks. The second mailing included an anchor question on the global rating of change, in which patients could indicate if their symptoms had changed over the last two weeks. Only patients who reported that their symptoms had not changed were included in the repeatability analysis. If returned questionnaires were incomplete, several attempts were made to reach patients by telephone in order to complete the missing items. Data collection took place from April to June 2014.

### mPDQ-NL

The mPDQ-NL is a self-report questionnaire consisting of 12 items about neuropathic pain symptoms of the left or right knee or hip during the past week. The first item assesses the presence of pain radiation using a body map. The second item assesses the pain pattern, where patients have to choose between four figures representing distinctly described pain patterns. The following seven items rank pain quality on a 6-point Likert scale, 1 representing “never” and 5 representing “very strongly”. These items respectively assess *burning sensation*, *tingling or prickling sensation*, *pain at light touch*, *sudden pain attacks*, *pain at cold or warm stimulus*, *numbness* and *pain at light pressure*. The final three items rank pain intensity on a 0–10 numeric rating scale (NRS), where 0 represents “no pain” and 10 represents “excruciating pain”. These final three items are about “pain at this moment”, “worst pain in the past week” and “average pain in the past week”, respectively. The total score ranges from -1 to 38 points; the final three items on pain intensity are not included in the score. Cut-off points were chosen in accordance with the original PDQ [25]. A score ≤ 12 indicates a nociceptive pain profile and a score between 13–18 indicates a possible neuropathic pain profile. A score ≥19 indicates a likely neuropathic pain profile.

### Statistical Analysis

Statistical analyses were performed using IBM SPSS Statistics for Windows (version 22.0, IBM Corp., Armonk, NY). Statistical significance was considered to be indicated with a p-value < 0.05 (two-tailed). If patients reported no pain on all three NRS of the mPDQ-NL, they were excluded from the analyses. Patient characteristics were reported using descriptive statistics.

#### Internal consistency

Internal consistency reflects the extent to which items of a questionnaire measure the same concept. Factor analysis should first be conducted to determine whether the items of the questionnaire form one single overall dimension or more than one [28]. Exploratory factor analysis was conducted according to Kaiser’s criterion using varimax rotation. Cronbach’s alphas were subsequently calculated for each principal component that was found. Cronbach’s alphas were also calculated for the overall questionnaire and for the seven Likert items on pain quality, analogously to earlier studies [25,29,30]. A Crohnbach’s alpha of 0.70–0.95 is generally accepted as a measure of good internal consistency [28].

#### Repeatability

Repeatability concerns whether repeated measures in a stable person provide the same scores. Two parts are distinguished: agreement (represented by the absolute measurement error) and reliability (represented by the relative measurement error) [28,31].

Agreement reflects the extent to which the scores of repeated measures are close to each other [28]. Absolute measurement errors were determined by calculating the standard error of measurement (SEM). The SEM can be derived by dividing the standard deviation of the mean differences (SD_diff_) between baseline measurement and retest measurement by √ 2 [31]. The smallest “real” change that can be detected in scores, despite measurement error, is represented by the smallest detectable change (SDC). This SDC with a 95% confidence interval (CI) for an individual can be derived from the SEM by using the following formula: SDC_ind_ = 1.96 x SEM x √ 2. For a group the SDC with 95% CI can be calculated by using the following formula: SDC_group_ = 1.96 x SEM x √ 2 / √ n. Absolute agreement was assessed using the methods of Bland and Altman [32]. The mean difference was calculated between test and retest with a 95% CI. When zero lies inside this 95% CI of mean difference, agreement is considered to be achieved. Otherwise, when zero lies outside the 95% CI, bias between test and retest measurements might be present.

Reliability reflects the extent to which patients can be distinguished from each other, despite measurement errors [28]. Relative measurement errors were assessed using intraclass correlation coefficients (ICC) with a 95% CI. A two-way random effects model (type absolute agreement) was used. In general, an ICC above 0.70 is considered good [33].

#### Floor and ceiling effects

The percentages of patients with the highest and lowest possible scores on the total mPDQ-NL and on the individual pain quality items were calculated. If more than 15% of respondents have minimal or maximal scores, floor and ceiling effects are considered to be present [28]. This reduces reliability as participants with extreme scores cannot be distinguished from each other.

## Results

**Fig 1:**
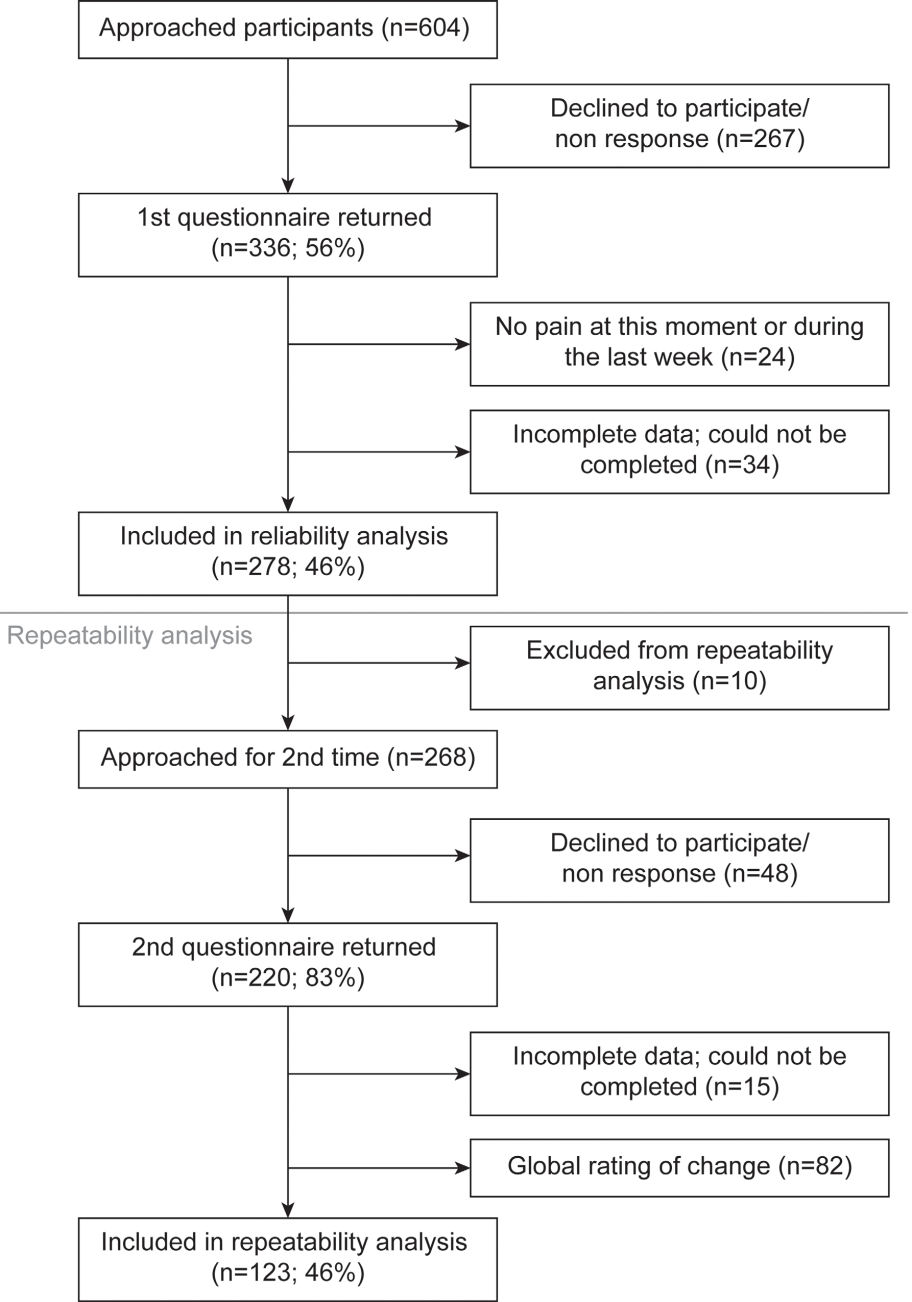
Flow chart showing inclusion procedure.

Translation of the mPDQ into Dutch was successful. No difficulties were encountered during pilot testing. In total, 604 patients were invited to participate in the reliability study. The response rate was 336 participants (55.6%). Of these respondents, 278 (46.0%) were included in the reliability study. Ten patients were excluded from the repeatability analysis. Reasons were because they received THA/TKA before the retest could be conducted or because the first questionnaire was returned with a considerable delay that surpassed the retest period. The response rate for the repeatability analysis was 220 participants (82.1%), 123 (45.9%) of whom were included in the repeatability analysis. Fig 1 shows the flow chart for inclusion. The number of cases lost because of missing items was 34 out of 336 respondents of the first questionnaire (10.1%) and 15 out of 220 respondents of the second questionnaire (6.8%).

Table 1 presents patient characteristics and mPDQ-NL scores for the study sample, with 17.7% of knee OA patients and 12.2% of hip OA patients showing a likely neuropathic pain profile (mPDQ-NL score ≥ 19), and 26.7% of knee OA patients and 23.7% of hip OA patients showing a possible neuropathic pain profile (mPDQ-NL score 13–18).

**Table 1:** Patient Characteristics.

Age (years)		65 ± 10 (37–90)
Gender	Female	162 (58.3%)
	Male	116 (41.7%)
Body Mass Index (kg/m^2^)		28 ± 5 (18–45)
Duration of pain (months) *		36 (18–78)
mPDQ-NL*		10 (7–16)

Abbreviations: mPDQ-NL, Dutch modified Pain DETECT Questionnaire. Mean ± SD (min-max) are shown for variables with normal distribution.

* Median (IQ range) is shown for variables with abnormal distribution. Gender is shown as number of patients (%).

### Clinimetric properties

#### Internal consistency

Exploratory factor analysis revealed two principal components with an eigenvalue ≥ 1. The first component had a Crohnbach’s alpha of 0.71, the second component 0.65. The general questionnaire showed a Crohnbach’s alpha of 0.77 and the seven Likert items on pain quality 0.80.

#### Repeatability

**Fig 2:**
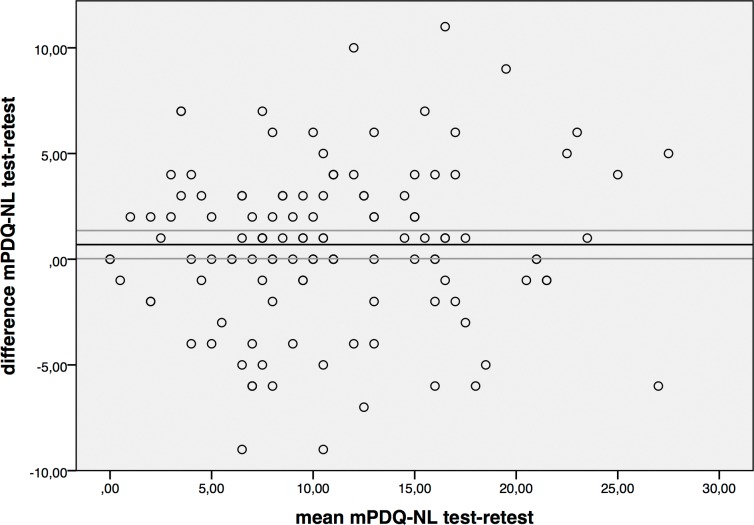
Bland Altman plot with 95% confidence interval (CI). Vertical axis: difference between mPDQ test and retest. Horizontal axis: mean mPDQ-NL when combining test and retest. The horizontal line represents the mean difference between test and retest. The gray lines represent the 95% CI of this mean difference. Notice that the 95% CI approaches 0.00.

Table 2 presents repeatability results for the mPDQ-NL. For total scores the SEM was 2.6, the SDC_ind_ 7.3 and the SDC_group_ 0.7. For individual items, SEM value ranges were 0.5–0.9, SDC_ind_ ranges 1.1–2.6 and SDC_group_ 0.1–0.3. For the total score, the mean difference between test and retest was 0.69 with a 95% CI of 0.03–1.36, which therefore did not contain zero. For individual items, the 95% CI contained zero for most individual items, except for *radiation* and *sudden pain attacks*. The Bland Altman plot is presented in Fig 2. For the total score, ICC was 0.90. For individual items ICC was high for most pain quality items (>0.70), except for *pain pattern* (ICC 0.52) and *radiation* (ICC 0.67).

**Table 2:** Repeatability.

	Baseline mean ± SD	Retest mean ± SD	Mean difference (95% CI)	SEM	SDC_ind_	SDC_grp_	ICC (95% CI)
Total score (n = 123)*	10.9 ± 6.5	10.5 ± 6.1	0.69 (1.36; 0.03)	2.6	7.3	0.7	0.90 (0.86–0.93)
Radiation (n = 129)*	1.4 ± 0.9	1.2 ± 1.0	0.28 (0.44; 0.12)	0.7	1.8	0.2	0.67 (0.53–0.77)
Pain pattern (n = 131)*	0.3 ± 0.7	0.3 ± 0.7	-0.01 (0.13; -0.15)	0.6	1.6	0.1	0.52 (0.32–0.66)
Burning sensation (n = 132)*	1.3 ± 1.5	1.3 ± 1.3	0.01 (0.22; -0.20)	0.9	2.4	0.3	0.76 (0.66–0.83)
Tingling/prickling sensation (n = 130)*	1.2 ± 1.5	1.4 ± 1.3	-0.19 (0.01; -0.40)	0.8	2.3	0.2	0.77 (0.68–0.84)
Pain at light touching (n = 131)*	0.9 ± 1.2	0.8 ± 1.0	0.12 (0.29; -0.04)	0.7	1.9	0.2	0.77 (0.67–0.84)
Sudden pain attacks/electric (n = 131)*	2.3 ± 1.6	2.1 ± 1.4	0.26 (0.49; 0.03)	0.9	2.6	0.2	0.75 (0.65–0.83)
Pain at cold/heat (n = 129)*	0.6 ± 1.0	0.6 ± 1.0	-0.03 (0.09; -0.16)	0.5	1.1	0.1	0.86 (0.80–0.90)
Numbness sensation (n = 130)*	1.1 ± 1.3	1.2 ± 1.3	-0.05 (0.15; -0.26)	0.9	2.4	0.2	0.74 (0.63–0.81)
Pain at slight pressure (n = 132)*	1.9 ± 1.5	1.8 ± 1.3	0.15 (0.33; -0.03)	0.8	2.1	0.2	0.84 (0.77–0.89)

Abbreviations: SD, standard deviation; CI, confidence interval; SEM, standard error of measurement; SDC_ind_, smallest detectable change at the individual level; SDC_grp_, smallest detectable change at the group level; ICC, intraclass correlation coefficient. Mean difference = baseline mean–retest mean.

* 123 complete cases were included in the repeatability analysis, yet for individual items there were fewer missing cases.

#### Floor and ceiling effects

For the total mPDQ-NL score, no floor or ceiling effects were found as no patient had the highest or lowest possible total score (-1 or 38 points). For all individual Likert items, >15% of patients had the lowest score, indicating that floor effects were present; no ceiling effects were perceived (Table 3).

**Table 3:** Floor and Ceiling effects.

	Burning sensation	Tingling/prickling sensation	Pain at light touching	Sudden pain attacks/electric	Pain at cold/heat	Numbness	Pain at slight pressure
0	122 **(43.9%)**	124 **(44.6%)**	145 **(52.2%)**	53 **(19.1%)**	185 **(66.5%)**	130 **(46.8%)**	67 **(24.1%)**
1	42 (15.1%)	49 (17.6%)	55 (19.8%)	33 (11.9%)	57 (20.5%)	50 (18.0%)	39 (14.0%)
2	35 (12.6%)	39 (14.0%)	34 (12.2%)	49 (17.6%)	14 (5.0%)	52 (18.7%)	57 (20.5%)
3	47 (16.9%)	35 (12.6%)	31 (11.2%)	63 (22.7%)	17 (6.1%)	27 (9.7%)	59 (21.2%)
4	26 (9.4%)	25 (9.0%)	11 (4.0%)	57 (20.5%)	3 (1.1%)	13 (4.7%)	49 (17.6%)
5	6 **(2.2%)**	6 **(2.2%)**	2 **(0.7%)**	23 **(8.3%)**	2 **(0.7%)**	6 **(2.2%)**	7 **(2.5%)**

Number of patients per item score (%). Total n = 278

## Discussion

This study is the first to provide a Dutch mPDQ to fit hip and knee OA patients, as well as to assess internal consistency, reliability and agreement of the mPDQ hip and knee. Hence results from the present study can only be compared to reliability data of the PDQ, as no other studies on reliability of the mPDQ are available to our knowledge.

For internal consistency, Crohnbach’s alpha for the overall score was 0.77, which is considered good [28]. This score is comparable to the Japanese, Turkish and Spanish PDQs, where overall Crohnbach’s alphas of respectively 0.78, 0.81 and 0.86 were found. No Crohnbach’s alpha from the original PDQ was available for comparison. For the seven Likert items on pain quality, Crohnbach’s alpha was 0.80, which is comparable to the original, Japanese, Turkish and Spanish PDQs with values of respectively 0.83, 0.80, 0.80 and 0.89 [25,29,30,34]. To summarize, overall internal consistency of the mPDQ-NL knee and hip is good and internal consistency between the seven Likert items on pain quality is particularly good.

Repeatability was assessed in terms of agreement and reliability. For agreement, SEM seems acceptable with 2.6 points, as the mPDQ ranges from -1 to 38 points. The mPDQ-NL might be better at detecting changes between groups of patients than at the individual level, considering that the individual SDC in the overall score was 7.3 points and the SDC at the group level was 0.7 points. However, to properly assess the value of these SDC figures they should be compared to the minimal important change (MIC), which could not be determined from the current data.

When assessing agreement according to Bland and Altman, it should be mentioned that the 95% CI did not contain zero for the overall mPDQ-NL score, although it almost reached zero (0.03–1.36). This means that a systematic measurement bias between the test and retest might be present. Even though only subjects who reported that their pain had not changed over the last two weeks were included in the analysis, the overall mPDQ-NL values were consistently slightly lower at retest. Patients may have had some difficulty estimating the global rate of change of the pain they experienced in the previous two weeks. The Bland and Altman plot shows an equal scatter of the difference in mPDQ-NL though, which means that no actual trend can be observed (Fig 2).

Reliability of the mPDQ-NL was good (ICC: 0.90), so it seems able to distinguish patients from each other despite measurement error. This is in line with previous PDQ reliability studies. For the Japanese PDQ an overall ICC of 0.94 was found, but only 11 subjects were included in their repeatability analysis. For the Spanish PDQ a repeatability analysis was conducted on 26 patients and an overall ICC of 0.93 was found.

No significant floor or ceiling effects were encountered for total mPDQ-NL scores. However, floor effects were found for all the individual Likert items. As only a subset of knee and hip OA patients experience neuropathic pain symptoms, it is understandable that floor effects were found. Considering that these floor effects for individual Likert items were not represented in the overall mPDQ-NL scores, it is likely that patients with subtle neuropathic symptoms can be distinguished from each other. This is important for the mPDQ-NL in order to identify subtle neuropathic symptoms within the broader spectrum of OA symptoms.

Because the mPDQ-NL considers symptoms other than actual pain (such as *numbness*), it is possible for patients with a low NRS for general pain intensity to reach a considerable mPDQ-NL score. Therefore, if patients reported even the least amount of pain (NRS >0) they were included in the study in order to evaluate neuropathic pain for the total range of symptomatic OA patients. Even in this population sample consisting of patients with different degrees of OA pain, 35.9% of hip OA patients and 44.4% of knee OA patients suffer from possible or likely neuropathic pain. The magnitude of these figures might support current literature on the importance of sensitization in OA pain [3,7,8,11,13]. Identification of patients with neuropathic features could facilitate customizing their treatment, for example by offering neuromodulating medication such as duloxetine. In the setting of future research, identifying subgroups of OA patients with neuropathic features could help assess the true effectiveness of these neuromodulating medications [10].

One of the strengths of the present study is that international guidelines were followed in the translation process. Furthermore, this study has ample sample size. When compared to other studies there is a relevant and recommended time interval between test and retest. Patients that reported a change in their pain over the two weeks between test and retest were excluded from the repeatability analysis [29,30,35]. The percentage of cases lost to analysis due to missing items was <15%, therefore selection bias is unlikely and the results can be considered generalizable to the missing part of the population [36]. To our knowledge, the present study is the first to clearly distinguish between reliability and agreement when assessing repeatability as recommended by COSMIN [28]. Although this study provides SEM and SDCs, which give some indication of evaluative purposes, future studies are needed to relate these values to the MIC and assess responsiveness. Validity of the mPDQ-NL also needs to be investigated in future studies on the construct and the factor structure of the mPDQ-NL.

## Conclusion

The mPDQ was successfully translated and adapted to fit Dutch hip and knee OA patients. It shows good internal consistency and small relative measurement errors, represented by a good ICC. Absolute measurement error, represented by the SEM, was acceptable. However, a measurement bias might be present. Based on this study, the mPDQ-NL seems to be fit as a discriminative tool to identify knee and hip OA patients with a neuropathic pain profile. Whether it is also suitable as an evaluative tool to record changes over time or changes after an intervention remains to be investigated further. As OA is the most common joint disease with a great burden on healthcare worldwide, it is of the utmost importance to optimize its treatment options. In order to do this, pain in OA patients needs to be clarified further. The mPDQ-NL hip and knee could facilitate in this quest by identifying knee and hip OA patients with a neuropathic pain profile.

## Supporting Information

S1 Database (SAV)

S1 Text mPDQ-NL hip and knee. (PDF)
