# Supplementary material for: The Modified painDETECT Questionnaire for Patients with Hip or Knee Osteoarthritis: Translation into Dutch, Cross-Cultural Adaptation and Reliability Assessment
Source: PLoS One. 2015 Dec 31;10(12):e0146117. doi: 10.1371/journal.pone.0146117 (PMC4697818; doi:10.1371/journal.pone.0146117)
Supplement: S1 Text — (PDF) [file pone.0146117.s002.pdf]

Modified painDETECT

We hebben gemerkt dat mensen verschillende woorden gebruiken om verschillende soorten van pijn bij artrose te beschrijven.
Naast pijn beschrijven sommige mensen vervelende klachten zoals zeurende pijn, stijfheid en zelfs tintelingen rondom hun gewrichten. Vanaf nu gebruiken we simpelweg het woord "pijn" voor *alle* vervelende klachten.

Deze vragenlijst vraagt naar klachten van artrose van de heup. We zullen hier vragen naar klachten in **rust**, niet naar uw klachten tijdens lichamelijke activiteit.
De gegeven beschrijvingen kunnen, maar hoeven niet overeen te komen met uw "pijn", ongeacht hoe ernstig deze is. Laat alstublieft geen enkele vraag onbeantwoord, zodat we zeker kunnen zijn over uw symptomen.

De vragenlijst is opgedeeld in 2 delen. Deel 1 vraagt naar uw RECHTER heup. Deel 2 vraagt naar uw LINKER heup.

Deel 1: RECHTER HEUP

Denkt u alstublieft ALLEEN aan uw **RECHTER** HEUP wanneer u de volgende vragen beantwoordt.

1. Straalde de "pijn" van uw RECHTER heup **naar boven of naar beneden** uit over uw RECHTER been in de **AFGELOPEN WEEK**?
   (Vink (√) hieronder JA of NEE aan)

JA ☐ → *Zo " JA "*, **markeer** alstublieft de "pijnlijke" gebieden van uw RECHTER heup op het hiernaast staande plaatje

NEE ☐ → *Als u "NEE"* heeft aangevinkt, ga dan op de volgende bladzijde verder met Vraag 2.

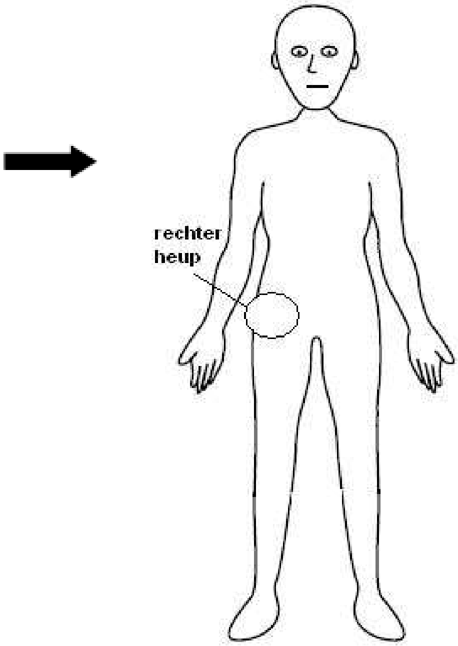

2. Kruis één afbeelding aan die het verloop van de “pijn” aan uw RECHTER heup in de **AFGELOPEN WEEK** het beste weergeeft: (Vink (√) één hokje aan die het beste bij uw pijn past)

☐ 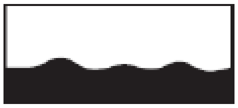 Aanhoudende pijn met lichte schommelingen

☐ 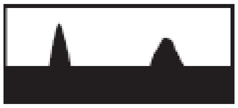 Aanhoudende pijn met pijnaanvallen

☐ 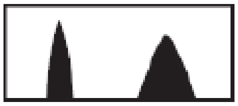 Pijnaanvallen, daartussen pijnvrij

☐ 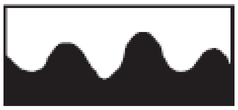 Pijnaanvallen, daartussen pijn

☐ Niet van toepassing (geen pijn)

3. Kies alstublieft het antwoord dat het beste de aard van de “pijn” aan uw RECHTER heup in de **AFGELOPEN WEEK** beschrijft. Denk eraan dat we het woord ‘pijn’ gebruiken voor alle vervelende klachten die u zou kunnen beschrijven als pijn. **Laat u alstublieft geen vragen onbeantwoord**.

(Vink (√) één hokje aan die het beste bij uw klacht past)

	**Nooit**	**Vrijwel niet**	**Licht**	**Matig**	**Sterk**	**Zeer Sterk**
**a. Hebt u last van een branderig gevoel (bijvoorbeeld als van brandnetels) in of rondom uw RECHTER heup?**	☐	☐	☐	☐	☐	☐
**b. Hebt u een kriebelend of prikkelend gevoel in het pijnlijke gebied van uw RECHTER heup (zoals mieren, zwakke stroom)?**	☐	☐	☐	☐	☐	☐

3. Aard van de “pijn” aan uw RECHTER heup in de **AFGELOPEN WEEK** (vervolg)

	**Nooit**	**Vrijwel niet**	**Licht**	**Matig**	**Sterk**	**Zeer Sterk**
**c. Is een lichte aanraking (kleding, beddengoed) in dit gebied pijnlijk?**	☐	☐	☐	☐	☐	☐
**d. Hebt u <u>schietende</u> pijnaanvallen in het pijnlijke gebied van uw RECHTER heup, als elektrische scheuten?**	☐	☐	☐	☐	☐	☐
**e. Is <u>kou of warmte</u> (badwater) in dit gebied wel eens pijnlijk?**	☐	☐	☐	☐	☐	☐
**f. Hebt u last van een doof gevoel in of rondom uw RECHTER heup?**	☐	☐	☐	☐	☐	☐
**g. Wekt een lichte <u>druk,</u> bijvoorbeeld met de vinger, in dit gebied pijn op?**	☐	☐	☐	☐	☐	☐

4. In de volgende paar vragen kunt u ons vertellen over <u>de mate</u> van ongemak die u ervaart in of rondom uw RECHTER heup

a. Welk cijfer geeft u op dit moment de pijn aan uw RECHTER heup op een 0-10 schaal. 0 staat voor ‘geen pijn’ en 10 is ‘de ergste pijn voorstelbaar’ (Omcirkel slechts EEN nummer)

Geen ‘pijn’ **Ergste ‘pijn’ voorstelbaar**

0	1	2	3	4	5	6	7	8	9	10

b. Hoe intens was de **HEFTIGSTE** ‘pijn’ in de **AFGELOPEN WEEK** aan uw RECHTER heup op een schaal van 0-10. 0 staat voor ‘geen pijn’ en 10 is ‘de ergste pijn voorstelbaar’ (Omcirkel slechts EEN nummer)

Geen ‘pijn’ **Ergste ‘pijn’ voorstelbaar**

0	1	2	3	4	5	6	7	8	9	10

c. Hoe intens was **GEMIDDELD** de ‘pijn’ in de **AFGELOPEN WEEK** aan uw RECHTER heup op een schaal van 0-10. 0 staat voor ‘geen pijn’ en 10 is ‘de ergste pijn voorstelbaar’ (Omcirkel slechts EEN nummer)

Geen ‘pijn’ **Ergste ‘pijn’ voorstelbaar**

0	1	2	3	4	5	6	7	8	9	10

Deel 2: LINKER HEUP

Denkt u alstublieft ALLEEN aan uw **LINKER** HEUP wanneer u de volgende vragen beantwoordt.

1. Straalde de “pijn” van uw LINKER heup **naar boven of naar beneden** uit over uw LINKER been in de **AFGELOPEN WEEK**?
   (Vink (√) hieronder JA of NEE aan)

JA ☐ → *Zo “ JA “*, **markeer** alstublieft de “pijnlijke” gebieden van uw LINKER heup op het hiernaast staande plaatje

NEE ☐ → *Als u “NEE”* heeft aangevinkt, ga dan op de volgende bladzijde verder met Vraag 2.

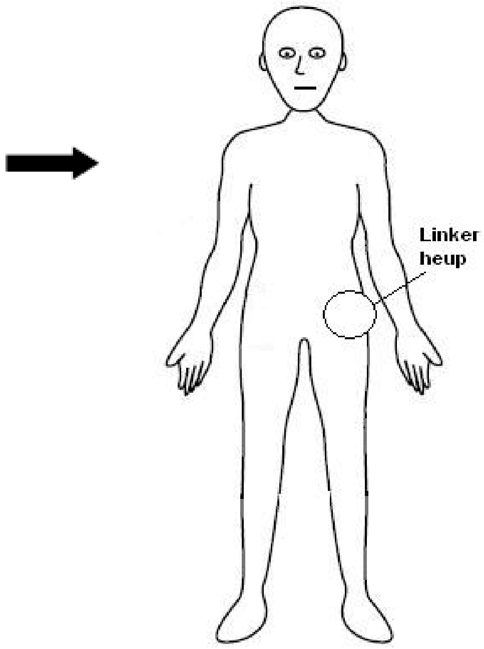

2. Kruis één afbeelding aan die het verloop van de “pijn” aan uw LINKER heup in de **AFGELOPEN WEEK** het beste weergeeft: (Vink (√) één hokje aan die het beste bij uw pijn past)

☐ 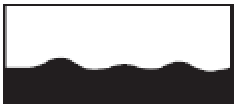 Aanhoudende pijn met lichte schommelingen

☐ 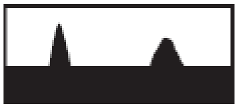 Aanhoudende pijn met pijnaanvallen

☐ 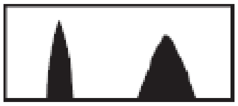 Pijnaanvallen, daartussen pijnvrij

☐ 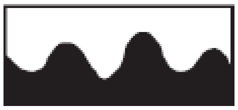 Pijnaanvallen, daartussen pijn

☐ ▭ Niet van toepassing (geen pijn)

3. Kies alstublieft het antwoord dat het beste de aard van de “pijn” aan uw LINKER heup in de **AFGELOPEN WEEK** beschrijft.
Denk eraan dat we het woord ‘pijn’ gebruiken voor alle vervelende klachten die u zou kunnen beschrijven als pijn. **Laat u alstublieft geen vragen onbeantwoord**.

(Vink (√) één hokje aan die het beste bij uw klacht past)

	**Nooit**	**Vrijwel niet**	**Licht**	**Matig**	**Sterk**	**Zeer Sterk**
**a. Hebt u last van een branderig gevoel (bijvoorbeeld als van brandnetels) in of rondom uw LINKER heup?**	☐	☐	☐	☐	☐	☐
**b. Hebt u een kriebelend of prikkelend gevoel in het pijnlijke gebied van uw LINKER heup (zoals mieren, zwakke stroom)?**	☐	☐	☐	☐	☐	☐

3. Aard van de “pijn” aan uw LINKER heup in de **AFGELOPEN WEEK** (vervolg)

	**Nooit**	**Vrijwel niet**	**Licht**	**Matig**	**Sterk**	**Zeer Sterk**
**c. Is een lichte aanraking (kleding, beddengoed) in dit gebied pijnlijk?**	☐	☐	☐	☐	☐	☐
**d. Hebt u schietende pijnaanvallen in het pijnlijke gebied van uw LINKER heup, als elektrische scheuten?**	☐	☐	☐	☐	☐	☐
**e. Is kou of warmte (badwater) in dit gebied wel eens pijnlijk?**	☐	☐	☐	☐	☐	☐
**f. Hebt u last van een doof gevoel in of rondom uw LINKER heup?**	☐	☐	☐	☐	☐	☐
**g. Wekt een lichte druk, bijvoorbeeld met de vinger, in dit gebied pijn op?**	☐	☐	☐	☐	☐	☐

4. In de volgende paar vragen kunt u ons vertellen over de mate van ongemak die u ervaart in of rondom uw LINKER heup

a. Welk cijfer geeft u op dit moment de pijn aan uw LINKER heup op een 0-10 schaal. 0 staat voor ‘geen pijn’ en 10 is ‘de ergste pijn voorstelbaar’ (Omcirkel slechts EEN nummer)

Geen ‘pijn’ **Ergste ‘pijn’ voorstelbaar**

0	1	2	3	4	5	6	7	8	9	10

b. Hoe intens was de **HEFTIGSTE** ‘pijn’ in de **AFGELOPEN WEEK** aan uw LINKER heup op een schaal van 0-10. 0 staat voor ‘geen pijn’ en 10 is ‘de ergste pijn voorstelbaar’ (Omcirkel slechts EEN nummer)

Geen ‘pijn’ **Ergste ‘pijn’ voorstelbaar**

0	1	2	3	4	5	6	7	8	9	10

c. Hoe intens was **GEMIDDELD** de ‘pijn’ in de **AFGELOPEN WEEK** aan uw LINKER heup op een schaal van 0-10. 0 staat voor ‘geen pijn’ en 10 is ‘de ergste pijn voorstelbaar’ (Omcirkel slechts EEN nummer)

Geen ‘pijn’ **Ergste ‘pijn’ voorstelbaar**

0	1	2	3	4	5	6	7	8	9	10

Modified painDETECT

We hebben gemerkt dat mensen verschillende woorden gebruiken om verschillende soorten van pijn bij artrose te beschrijven.
Naast pijn beschrijven sommige mensen vervelende klachten zoals zeurende pijn, stijfheid en zelfs tintelingen rondom hun gewrichten. Vanaf nu, gebruiken we simpelweg het woord “pijn” voor *alle* vervelende klachten.

Deze vragenlijst vraagt naar klachten van artrose van de knie. We zullen hier vragen naar klachten in rust, niet naar uw klachten tijdens lichamelijke activiteit.
De gegeven beschrijvingen kunnen, maar hoeven niet overeen te komen met uw “pijn”, ongeacht hoe ernstig deze is. Laat alstublieft geen enkele vraag onbeantwoord, zodat we zeker kunnen zijn over uw symptomen.

De vragenlijst is opgedeeld in 2 delen. Deel 1 vraagt naar uw RECHTER knie. Deel 2 vraagt naar uw LINKER knie.

Deel 1: RECHTER KNIE

Denkt u alstublieft ALLEEN aan uw **RECHTER** KNIE wanneer u de volgende vragen beantwoordt.

1. Straalde de “pijn” van uw RECHTER knie **naar boven of naar beneden** uit over uw RECHTER been in de **AFGELOPEN WEEK**?
   (Vink (√) hieronder JA of NEE aan)

JA ☐ → *Zo “ JA “*, **markeer** alstublieft de “pijnlijke” gebieden van uw RECHTER been op het hiernaast staande plaatje

NEE ☐ → *Als u “NEE”* heeft aangevinkt, ga dan op de volgende bladzijde verder met Vraag 2.

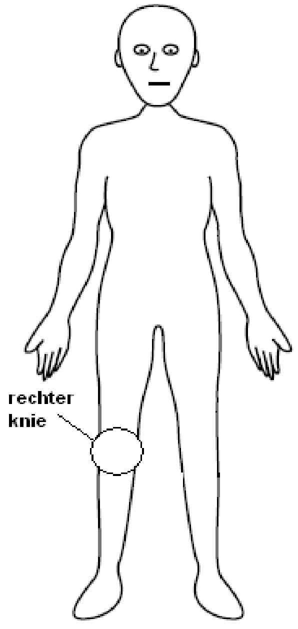

5. Kruis één afbeelding aan die het verloop van de “pijn” aan uw RECHTER knie in de **AFGELOPEN WEEK** het beste weergeeft: (Vink (√) één hokje aan die het beste bij uw pijn past)

☐	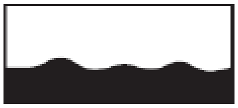	Aanhoudende pijn met lichte schommelingen
☐	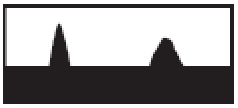	Aanhoudende pijn met pijnaanvallen
☐	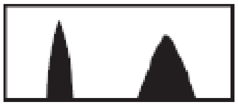	Pijnaanvallen, daartussen pijnvrij
☐	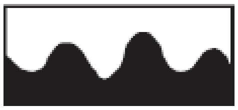	Pijnaanvallen, daartussen pijn
☐	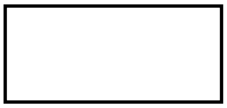	Niet van toepassing (geen pijn)

6. Kies alstublieft het antwoord dat het beste de aard van de “pijn” aan uw RECHTER knie in de **AFGELOPEN WEEK** beschrijft. Denk eraan dat we het woord ‘pijn’ gebruiken voor alle vervelende klachten die u zou kunnen beschrijven als pijn. **Laat u alstublieft geen vragen onbeantwoord**.

(Vink (√) één hokje aan die het beste bij uw klacht past)

	**Nooit**	**Vrijwel niet**	**Licht**	**Matig**	**Sterk**	**Zeer Sterk**
**a. Hebt u last van een branderig gevoel (bijvoorbeeld als van brandnetels) in of rondom uw RECHTER knie?**	☐	☐	☐	☐	☐	☐
**b. Hebt u een kriebelend of prikkelend gevoel in het pijnlijke gebied van uw RECHTER knie (zoals mieren, zwakke stroom)?**	☐	☐	☐	☐	☐	☐

3. Aard van de “pijn” aan uw RECHTER knie in de **AFGELOPEN WEEK** (vervolg)

	**Nooit**	**Vrijwel niet**	**Licht**	**Matig**	**Sterk**	**Zeer Sterk**
**c. Is een lichte aanraking (kleding, beddengoed) in dit gebied pijnlijk?**	☐	☐	☐	☐	☐	☐
**d. Hebt u <u>schietende</u> pijnaanvallen in het pijnlijke gebied van uw RECHTER knie, als elektrische scheuten?**	☐	☐	☐	☐	☐	☐
**e. Is <u>kou of warmte</u> (badwater) in dit gebied wel eens pijnlijk?**	☐	☐	☐	☐	☐	☐
**f. Hebt u last van een doof gevoel in of rondom uw RECHTER knie?**	☐	☐	☐	☐	☐	☐
**g. Wekt een lichte <u>druk,</u> bijvoorbeeld met de vinger, in dit gebied pijn op?**	☐	☐	☐	☐	☐	☐

7. In de volgende paar vragen kunt u ons vertellen over <u>de mate</u> van ongemak die u ervaart in of rondom uw RECHTER knie

d. Welk cijfer geeft u op dit moment de pijn aan uw RECHTER knie op een 0-10 schaal. 0 staat voor ‘geen pijn’ en 10 is ‘de ergste pijn voorstelbaar’ (Omcirkel slechts EEN nummer)

Geen ‘pijn’ **Ergste ‘pijn’ voorstelbaar**

0	1	2	3	4	5	6	7	8	9	10

e. Hoe intens was de **HEFTIGSTE** ‘pijn’ in de **AFGELOPEN WEEK** aan uw RECHTER knie op een schaal van 0-10. 0 staat voor ‘geen pijn’ en 10 is ‘de ergste pijn voorstelbaar’ (Omcirkel slechts EEN nummer)

Geen ‘pijn’ **Ergste ‘pijn’ voorstelbaar**

0	1	2	3	4	5	6	7	8	9	10

f. Hoe intens was **GEMIDDELD** de ‘pijn’ in de **AFGELOPEN WEEK** aan uw RECHTER knie op een schaal van 0-10. 0 staat voor ‘geen pijn’ en 10 is ‘de ergste pijn voorstelbaar’ (Omcirkel slechts EEN nummer)

Geen ‘pijn’ **Ergste ‘pijn’ voorstelbaar**

0	1	2	3	4	5	6	7	8	9	10

Deel 2: LINKER KNIE

Denkt u alstublieft ALLEEN aan uw **LINKER** KNIE wanneer u de volgende vragen beantwoordt.

1. Straalde de “pijn” van uw LINKER knie **naar boven of naar beneden** uit over uw LINKER been in de **AFGELOPEN WEEK**?
   (Vink (√) hieronder JA of NEE aan)

JA ☐ → *Zo “ JA “*, **markeer** alstublieft de “pijnlijke” gebieden van uw LINKER been op het hiernaast staande plaatje

NEE ☐ → *Als u “NEE”* heeft aangevinkt, ga dan op de volgende bladzijde verder met Vraag 2.

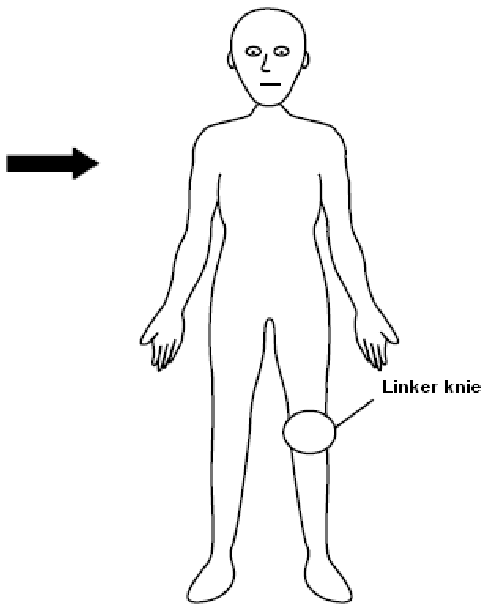

2. Kruis één afbeelding aan die het <u>verloop</u> van de “pijn” aan uw LINKER knie in de **AFGELOPEN WEEK** het beste weergeeft: (Vink (√) één hokje aan die het beste bij uw pijn past)

☐ 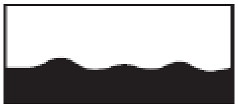 Aanhoudende pijn met lichte schommelingen

☐ 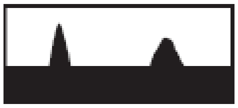 Aanhoudende pijn met pijnaanvallen

☐ 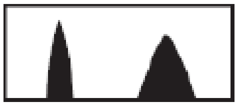 Pijnaanvallen, daartussen pijnvrij

☐ 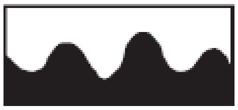 Pijnaanvallen, daartussen pijn

☐ Niet van toepassing (geen pijn)

3. Kies alstublieft het antwoord dat het beste de aard van de “pijn” aan uw LINKER knie in de **AFGELOPEN WEEK** beschrijft.
Denk eraan dat we het woord ‘pijn’ gebruiken voor alle <u>vervelende klachten</u> die u zou kunnen beschrijven als pijn. **Laat u alstublieft geen vragen onbeantwoord**.

(Vink (√) één hokje aan die het beste bij uw klacht past)

	**Nooit**	**Vrijwel niet**	**Licht**	**Matig**	**Sterk**	**Zeer Sterk**
**a. Hebt u last van een <u>branderig</u> gevoel (bijvoorbeeld als van brandnetels) in of rondom uw LINKER knie?**	☐	☐	☐	☐	☐	☐
**b. Hebt u een kriebelend of prikkelend gevoel in het pijnlijke gebied van uw LINKER knie (zoals mieren, zwakke stroom)?**	☐	☐	☐	☐	☐	☐

3. Aard van de “pijn” aan uw LINKER knie in de **AFGELOPEN WEEK** (vervolg)

	**Nooit**	**Vrijwel niet**	**Licht**	**Matig**	**Sterk**	**Zeer Sterk**
**c. Is een lichte aanraking (kleding, beddengoed) in dit gebied pijnlijk?**	☐	☐	☐	☐	☐	☐
**d. Hebt u schietende pijnaanvallen in het pijnlijke gebied van uw LINKER knie, als elektrische scheuten?**	☐	☐	☐	☐	☐	☐
**e. Is kou of warmte (badwater) in dit gebied wel eens pijnlijk?**	☐	☐	☐	☐	☐	☐
**f. Hebt u last van een doof gevoel in of rondom uw LINKER knie?**	☐	☐	☐	☐	☐	☐
**g. Wekt een lichte druk, bijvoorbeeld met de vinger, in dit gebied pijn op?**	☐	☐	☐	☐	☐	☐

4. In de volgende paar vragen kunt u ons vertellen over de mate van ongemak die u ervaart in of rondom uw LINKER knie

d. Welk cijfer geeft u op dit moment de pijn aan uw LINKER knie op een 0-10 schaal. 0 staat voor ‘geen pijn’ en 10 is ‘de ergste pijn voorstelbaar’ (Omcirkel slechts EEN nummer)

Geen ‘pijn’ **Ergste ‘pijn’ voorstelbaar**

0	1	2	3	4	5	6	7	8	9	10

e. Hoe intens was de **HEFTIGSTE** ‘pijn’ in de **AFGELOPEN WEEK** aan uw LINKER knie op een schaal van 0-10. 0 staat voor ‘geen pijn’ en 10 is ‘de ergste pijn voorstelbaar’ (Omcirkel slechts EEN nummer)

Geen ‘pijn’ **Ergste ‘pijn’ voorstelbaar**

0	1	2	3	4	5	6	7	8	9	10

f. Hoe intens was **GEMIDDELD** de ‘pijn’ in de **AFGELOPEN WEEK** aan uw LINKER knie op een schaal van 0-10. 0 staat voor ‘geen pijn’ en 10 is ‘de ergste pijn voorstelbaar’ (Omcirkel slechts EEN nummer)

Geen ‘pijn’ **Ergste ‘pijn’ voorstelbaar**

0	1	2	3	4	5	6	7	8	9	10
